# Cognitive segmentation and fluid reasoning in childhood

**DOI:** 10.1177/17470218221116054

**Published:** 2022-08-24

**Authors:** Sinéad O’Brien, Daniel J Mitchell, John Duncan, Joni Holmes

**Affiliations:** 1 MRC Cognition and Brain Sciences Unit, University of Cambridge, Cambridge, UK; 2 College of Staten Island, The City University of New York, New York, NY, USA; 3 Department of Experimental Psychology, University of Oxford, Oxford, UK; 4 School of Psychology, University of East Anglia, Norwich, UK

**Keywords:** Cognitive segmentation, fluid intelligence, problem-solving, analogical reasoning, matrix reasoning

## Abstract

The ability to solve novel complex problems predicts success in a wide range of areas. Recent research suggests that the ability to cognitively segment complex problems into smaller parts constrains nonverbal reasoning in adults. This study aimed to test whether cognitively segmenting problems improves nonverbal reasoning performance for children as it does for adults. A total of 115 children aged 6–10 years completed two versions of a modified traditional matrix reasoning task in which demands on working memory, integration, and processing speed were minimised, such that the only significant requirement was to break each problem into its constituent parts. In one version of the task, participants were presented with a traditional 2×2 Matrix and asked to draw the missing matrix item into a response box below. In a second version, the problem was broken down into its component features across three separate cells, reducing the need for participants to segment the problem. As with adults, performance was better in the condition in which the problems were separated into component parts. Children with lower fluid intelligence did not benefit more in the separated condition than children with higher fluid intelligence, and there was no evidence that segmenting problems was more beneficial for younger than older children. This study demonstrates that cognitive segmentation is a critical component of complex problem-solving for children, as it is for adults. By forcing children to focus their attention on separate parts of a complex visual problem, their performance can be dramatically improved.

## Introduction

Fluid intelligence describes the ability to solve complex problems under novel conditions where learned skills and knowledge are minimised (Cattell, 1971). Early fluid reasoning skills predict later educational achievement, employment prospects, and both mental and physical health outcomes (Chen et al., 2017; Batty & Deary, 2004; Deary, 2012; Duncan et al., 2020; Postlethwaite, 2011; Sternberg et al., 2001; Zammit et al., 2004). Cognitive processes commonly implicated in fluid reasoning include working memory (Colom et al., 2008; Engle, 2010; Kyllonen & Christal, 1990) and processing speed (Jungeblut et al., 2021; Salthouse, 1996). More recently, Duncan (2013) proposed that cognitive segmentation, the ability to separate a complex problem into component parts, is also vital for the successful completion of fluid reasoning tasks. Duncan et al. demonstrated that modifying the layout of a traditional fluid reasoning task to aid cognitive segmentation improved task performance in adults, particularly for those with low fluid intelligence (Duncan et al., 2017). This study builds on Duncan et al.’s (2017) work to test whether cognitive segmentation improves children’s performance on complex fluid reasoning problems in the same way as it does for some adults.

### Matrix reasoning

Performance on fluid reasoning tasks is highly positively correlated with scores on tests of other cognitive abilities—something often referred to as the positive manifold (Deary, 2012; Jensen, 1998; Spearman, 1927). There are various theories of the positive manifold (e.g., Kovacs & Conway, 2016; Van Der Maas et al., 2006), but the dominant account suggests that it reflects an underlying latent factor of general cognitive ability (e.g., Spearman, 1904). This might reflect individual differences in working memory capacity (Kyllonen & Christal, 1990; Tourva et al., 2016), the speed of information processing (Detterman, 2002; Jensen, 2006; Salthouse, 1996), or the ability to decompose problems into manageable parts (Duncan et al., 2017).

**Figure 1.**
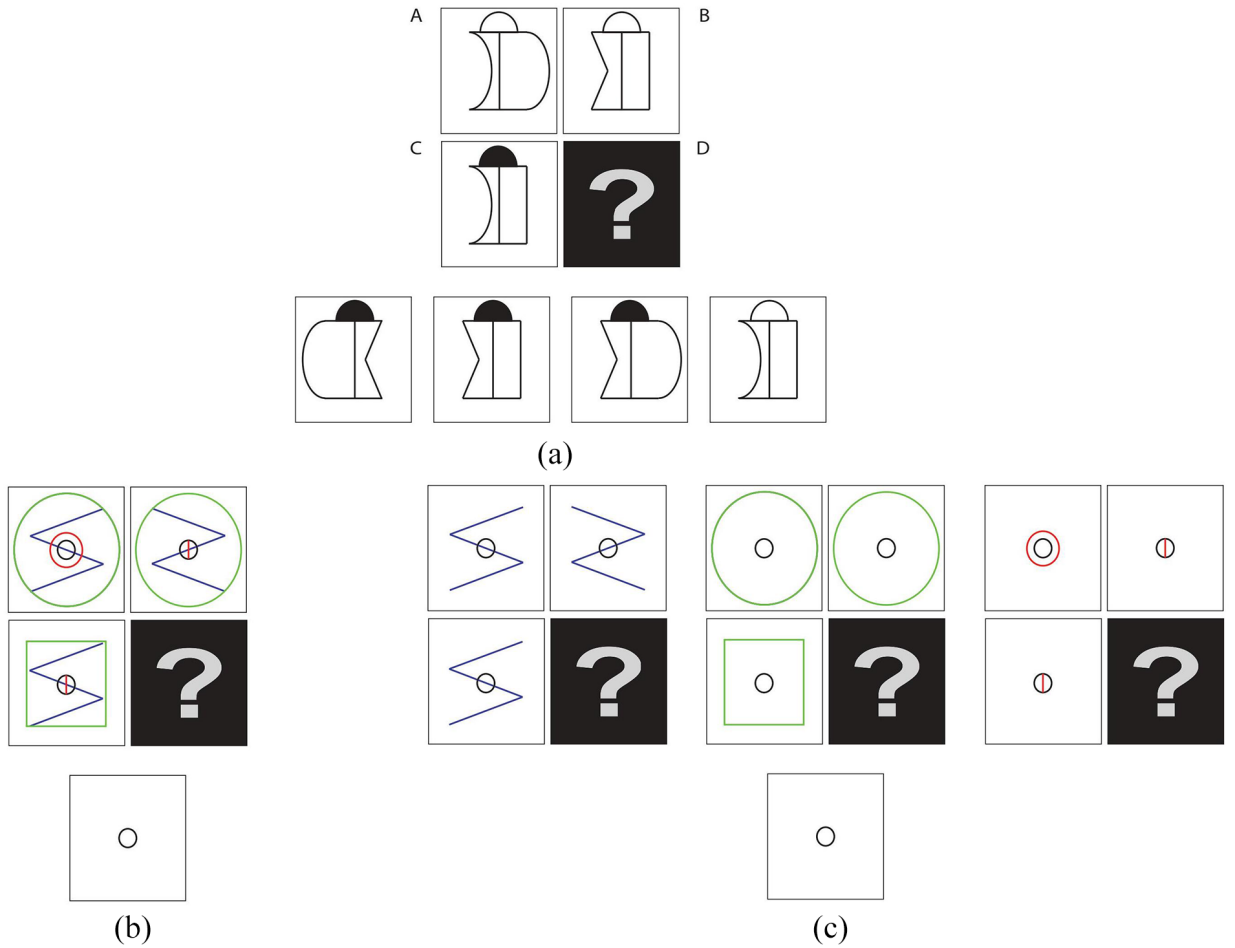
(a) Example of a traditional matrix reasoning task item. (b) Cognitive segmentation combined item. (c) Cognitive segmentation separated item. Participants see cognitive segmentation task items in black and white. Features are shown in colour here for illustration purposes.

Fluid reasoning is often measured using tasks that require participants to identify abstract relations between elements and draw analogies to complete a pattern, because performance on such tasks has been found to be especially predictive of fluid intelligence. For example, participants might be presented with a pair of related objects (A and B) alongside a third object (C). To solve the problem, participants must identify a fourth object (D) by working out what rule relates A to B, and then applying this to C (A is to B as C is to D). In matrix reasoning tasks, the objects (geometric figures) are organised in matrices of 3×3 or 2×2 (see Figure 1a). The objects and their relations vary systematically across the rows and columns, with the bottom right cell of the matrix (cell D) left blank. Participants are typically required to select, from a set of options, which object fits the empty cell. The analogy can be solved by encoding the problem and creating a mental representation to identify features (rules and relationships) that connect the objects in the top row of the matrix (cells A and B: Figure 1a). These rules are then applied to the bottom row (cell C: Figure 1a) to create an appropriate representation to fill the blank cell (D: Figure 1a) (Primi, 2001; Sternberg, 1977). Thus matrix reasoning tasks, used to measure fluid intelligence, can be seen as a specific type of more general analogical reasoning tasks, with key features being that the objects are abstract shapes to minimise contributions of acquired semantic knowledge, and that the relationships can be described along multiple spatial axes: vertical (A:C and B:D), horizontal (A:B and C:D), or diagonal (A:D and B:C).

### Cognitive segmentation

Cognitive segmentation, the ability to separate complex problems into component parts that can be systematically attended to and processed, has long been described in the literature (Egeth, 1966; Luria & Tsvetkova, 1964; Nickerson, 1967), but until recently it has rarely been isolated and examined in relation to performance on nonverbal reasoning tasks. Duncan et al. (2017) modified a traditional matrix reasoning task, making segmentation easy or hard to achieve, while also reducing demands on working memory and processing speed. In traditional matrix reasoning tasks (Figure 1a), selecting the correct answer from a set of options requires multiple processes: identifying the individual features that connect the cells; establishing how they vary; holding in working memory different parts of the solution while working on others; and integrating different parts of the solution to choose between the possible answers, which may rely on processing speed to ensure all aspects of the problem are available simultaneously (Salthouse, 1996). Duncan et al. (2017) minimised the demands on speed, integration, and working memory by modifying 3-feature matrix reasoning problems such that the only significant requirement was to break the 3-feature problem into 1-feature parts: to focus on one soluble part at a time.

Adults completed two different versions of this modified task. In the Combined format (Figure 1b) participants were presented with a 2×2 Matrix and asked to draw the missing matrix item into a response box below. Drawing the solution (rather than selecting an answer from a set of options) allowed participants to focus on one feature at a time, reducing demands on integration and working memory, and the task was administered without time constraints in one experiment to eliminate processing speed demands. In the Separated format (Figure 1c) the problem was broken down into its component features across three separate cells, reducing the need for participants to segment the problem. Despite the reduced demands on working memory and processing speed, performance on the Combined version of the task remained poor for adults with low fluid intelligence. These errors largely vanished in the Separated condition when it became trivial to segment the problem into its component parts/features. These data indicate that when working memory, integration, and processing speed demands are reduced in matrix reasoning tasks, the ability to cognitively segment remains critical to success.

### Cognitive segmentation in children

Understanding whether children can spontaneously segment complex problems, and how segmentation develops, has important implications for classroom practice. The ability to break down complex problems is crucial for classroom learning and educational attainment: children need to be able to decompose multi-step instructions, and break individual learning tasks into their component parts for success (e.g., Jaroslawska et al., 2016). Knowing whether and when children can break down problems by themselves, and the beneficial effects of help in segmenting complex cognitive tasks, can provide a useful guide for teachers in terms of gauging the support they provide.

We propose that cognitive segmentation will constrain children’s nonverbal reasoning, and that the ability to cognitively segment develops with age. Children’s reasoning skills develop over time: children acquire the ability to represent and map relations both as their knowledge base grows (e.g., Gentner, 1988; Goswami & Brown, 1990) and their executive function skills develop (e.g., Morrison et al., 2006; Richland et al., 2006; Thibaut et al., 2010). Children also move through phases of matrix reasoning development before finally settling into a more consistent approach (Raven et al., 1983; Siegler & Svetina, 2002; Stevenson & Hickendorff, 2018; Vodegel Matzen et al., 1994).

In the least advanced phase, called the duplication or repetition phase, young children consistently duplicate a matrix item (e.g., the shape in cell C) irrespective of the complexity of the problem (Stevenson & Hickendorff, 2018). Children are also more likely to spend time focussing on the C cell than on cells A and B (Starr et al., 2018). Consistent with the literature using more semantic analogical reasoning tasks, in which young children aged up to 8 years orient to C and organise their search around this item (Thibaut & French, 2016), this suggests that young children have a tendency to focus on a single part of a problem.

In the “idiosyncratic phase” children use variable solution processes. Partial responses—choosing a response option with a partially correct solution—are common on easy items, whereas more complex items elicit varied errors (Stevenson & Hickendorff, 2018). The most common error in more advanced stages is almost exclusively arriving at a response option with a partially correct solution. This demonstrates more consistency in solution approaches with age (Raven et al., 1983; Siegler & Svetina, 2002; Vodegel Matzen et al., 1994).

Not all children progress sequentially through these phases of matrix reasoning development (Stevenson & Hickendorff, 2018). The strategies children use also vary, with differences between younger and older children and between children who are high or low performers. Eye- and mouse-tracking studies have identified two strategies used to solve analogies across different reasoning paradigms: constructive matching and response elimination (e.g., Bethell-Fox et al., 1984; Gonthier & Roulin, 2020). Constructive matching involves encoding and inferring the relationships between features, to create a mental representation of the solution, to compare with response alternatives. Response elimination involves the comparison of features between the matrix and response alternatives first, in order to eliminate options and select the correct solution. Children who use constructive matching perform better than those who use response elimination, and some individuals shift strategy to response elimination for more complex items (Chen et al., 2016; Niebaum & Munakata, 2021). This suggests that children shift from relying on response elimination strategies to increasingly using constructive matching.

Together these studies show that, with age, children take a more systematic approach to solving matrix reasoning problems. They are better able to focus on and encode each part of a problem to infer relations between the features in a matrix: they are potentially more able to segment problems into their constituent parts.

### The current study

The primary aim of this study is to extend Duncan et al.’s (2017) paradigm to children, to test whether segmenting problems into their constituent parts aids performance in the same way as it does for adults with low IQ. We hypothesised that children would perform better on the Separated than the Combined version of Duncan et al.’s (2017) modified matrix reasoning tasks. This is because children’s performance on complex tasks can be improved when “useful scaffolding” is used. That is, when others divide complex tasks into simpler more manageable parts (e.g., Duncan et al., 2021; Neitzel & Stright, 2003), and because breaking down matrix analogies into their component parts, by encouraging children to focus on separate parts of the problem through feature-by-feature feedback (Chen et al., 2016) or using constructed response formats (Denaes, 2012; Stevenson et al., 2016), improves performance.

Secondary aims of the study were to explore the effects of age (do younger children fail to segment more than older children?), and to test whether completing the Separated version of the modified matrix reasoning task first improves performance in a subsequent Combined condition. To our knowledge, this study presents the first attempt to develop a child-appropriate version of Duncan et al.’s (2017) task to isolate and examine the role of cognitive segmentation in relation to children’s fluid reasoning abilities. For this reason, we also explored the contribution of within task characteristics such as error rates, and drawing times to understand more about children’s problem-solving performance.

## Materials and methods

### Participants

A total of 115 participants aged 6–10 years (*M* = 104.79 months; *SD* = 13.36; 49.6% male) were recruited from four primary schools in the Republic of Ireland: 10 participants were recruited from School 1 (N of males: 6; age: *M* = 113.6; *SD* = 4.65); 39 participants from School 2 (N of males: 17; age: *M* = 101.85; *SD* = 14.38); 31 participants from School 3 (N of males: 18; age: *M* = 107.08; *SD* = 11.65); and 35 participants were recruited from School 4 (N of males: 16; age: *M* = 103.29; *SD* = 14.16). The participants recruited from these schools were from a range of socioeconomic backgrounds: affluent (10.4%); marginally above average (48.7%); marginally below average (35.7%); and disadvantaged (5.2%). Deprivation indices were retrieved from Pobal HP Deprivation Index (https://maps.pobal.ie/). Exclusion criteria were pre-existing genetic or neurological conditions. Parents/carers provided informed written consent and children provided assent. Children were not paid for their participation. Ethical approval was granted by the Cambridge Psychology Research Ethics Committee (CPREC reference: PRE.2018.051).

### Procedure

Each child completed three testing sessions. The two modified matrix reasoning tasks (Combined and Separated) were completed in Session 1, on a one-to-one basis in a quiet area of the child’s school. This session lasted approximately 40 min. An age-standardised test of fluid intelligence (Leiter-3; Roid et al., 2013) was administered in Session 2. Session 3 was conducted in groups of between 6 and 16 participants, in which participants completed a two-part fluid intelligence assessment on an iPad, based on subtests of the Cattell Culture Fair Test (Cattell, 1940; Institute for Personality and Ability Testing [IPAT], 1973a). Session order was fixed: all participants completed Session 1 first, followed 3–5 days later by Session 2, and 1–2 weeks later by Session 3.

### Design

This was a within-participants design: all participants completed all tasks. Task order was counterbalanced for the modified matrix reasoning tasks: 53.9% completed the Combined condition first and 46.1% completed the Separated condition first (Combined first: School 1 = 50%; School 2 = 56.4%; School 3 = 53.3%; School 4 = 52.8%). There was no significant difference in the age of the participants who completed the Combined condition first and those who completed the Separated condition first, *U* = 1,494.5, *p* = .39. Children completed one of four modified matrix reasoning task sets (A1, A2, B1 or B2, see “Measures” section below for details). Assignment to task order was counterbalanced by each child completing the next set on a rotating basis (e.g., Child 1 completed set A1, Child 2, set A2, Child 3 set B1, Child 4 set B2, Child 5 set A1, and so on). The subparts of the fluid intelligence tests were administered in fixed order.

### Measures

#### Fluid intelligence, based on Cattell Culture Fair test

Participants completed a two-part abstract reasoning assessment based on two subscales of the widely used Cattell Culture Fair tests (Scale 2, Form A: Cattell, 1940; IPAT, 1973a, 1973b). These were administered on an iPad within the Resilience in Education and Development App (RED App Ireland [version 1.3.4]: Bignardi et al., 2021). In Part 1, children were presented with 12 items, each containing a series of three abstract figures and one empty box. For each item, children were required to choose which of five abstract figures completed the series. In Part 2, children were presented with 14 items and asked to identify which of five abstract figures was different from the others for each item. Performance on both parts was measured as the proportion of correct responses out of the total number of items (Series: 12 items, Classification: 14 items). The average proportion of correct responses from the two subscales was used as a proxy measure of fluid reasoning.

#### Fluid intelligence, using the Leiter International Performance Scale-Third Edition (Leiter-3)

The Leiter-3 (Roid et al., 2013) was administered to provide an age-standardised measure of IQ. Participants completed four standardised subscales: Figure Ground; Form Completion, Classification Analogies, and Sequential Order. For each subscale, age-standardised scaled scores range from 0 to 20 (*M*: 10, *SD*: 3). The scaled scores from the four subscales are combined to form a Brief IQ score.

#### Modified matrix reasoning

Two modified matrix reasoning task conditions were administered: Combined and Separated. Unlike a traditional matrix reasoning task where participants select answers from multiple options, the children were asked to draw the answers in a box provided below each matrix for both tasks. The task was developed based on that used by Duncan et al. (2017) and adapted to be suitable for use with children.

Duncan et al. (2017) administered 22 items (2, 2-feature practice items, 10, 3-feature combined and separated test items) to adult participants across the two conditions. Each test item had three features, meaning it was composed of three geometric shapes. These features were attached to a common core (anchor) shape, which appeared in each cell of the matrix. This anchor shape was presented in each response box to facilitate drawing (see Figure 1b and c: the anchor shape is the small circle).

These items, and new ones developed using child-appropriate geometric shapes (Aslan & Arnas, 2007), were trialled with children aged 4–10 years. The purpose of this stimulus development and piloting stage was to ensure that children understood the task instructions, condition formats, and task items. While all children demonstrated an understanding of the task, children aged 5 years and younger struggled to complete the full task set. For this reason, children aged 6 years and above were recruited for the study.

Following piloting, 14 of the 3-feature items from Duncan et al. (2017) stimuli set were retained, 2 were modified slightly, and additional items were created (7, 1-feature practice items, 12, 2-feature practice items, and 4, additional 3-feature test items). The final task set thus included 19, 1- and 2-feature practice items, and 20, 3-feature test items, created in Adobe Illustrator CS6. Each test item had two formats: Combined (Figure 1b) and Separated (Figure 1c). The Combined format was similar to a traditional analogical reasoning task with each item presented in a 2×2 Matrix. In the Separated format, each feature of the item was presented in its own matrix. Unlike the traditional analogical reasoning task, where participants select answers from multiple options, the children were asked to draw the answers in a box provided below each matrix for both tasks. As in Duncan et al.’s (2017) study, an anchor shape was presented in each response box to facilitate the children’s drawing. Participants completed both modified matrix reasoning task conditions: Combined and Separated. Two items were used as practice trials. The remaining 20 items were split into two lists of 10 test items (Lists I and II), with a Combined and Separated version of each (e.g., Combined List I, Combined List II, Separated List I and Separated List II). These four lists were used to create 2, 20-item task sets with non-overlapping items in the Combined and Separated conditions: set A consisted of Combined items from List I and Separated items from List II; set B consisted of Combined items from List II and Separated item from List I. The presentation order of the items was counterbalanced: for half of set A and half of set B, the Combined items were presented first (A1 and B1) and for the other half the Separated items were presented first (A2 and B2). Children therefore completed one of four task sets (A1, A2, B1, or B2) containing 20 test items (10 Combined and 10 Separated).

Children completed two example items for 1-feature items. They were guided through these and feedback was given. They were then asked to complete 5, 1-feature practice items without feedback. For the 2-feature Combined condition, participants were led through 2, 2-feature example items and feedback was given. A full explanation about how to solve the items was provided by working through each example with the participants, one feature at a time. Although relations could be interpreted along horizontal, vertical, and diagonal axes of the matrix, in this experiment children were instructed to focus on relationships along the horizontal dimension. Participants were directed to one feature of the shape in cell A (e.g., a circle) and then the corresponding feature in cell B (e.g., another circle), and the experimenter explained how they were similar (e.g., “there is a circle in this box, and it has not changed in this box”). They were then asked to look at the corresponding feature in cell C (e.g., a square) and asked what they would expect in cell D. If they knew the answer (a square), they were asked to draw it in the response box. If the participant was not able to correctly solve the first feature, the correct response was provided, with an explanation about how to find it (e.g., “When we look across the top row, what has happened? The same must happen in the bottom boxes. First look at the circle on the left. In the top row, this shape does not change. So, in the bottom row the shape should not change either.”). Participants were then given another opportunity to draw the answer. These steps were then repeated for the second feature of the problem, and for both features of the second practice item. Having completed the example items, participants were then asked to complete 5, 2-feature Combined practice items on their own, without feedback. Before completing the 3-feature test items, participants were told “Now you will see puzzles with three shapes. Answer them in the same way as you did before.” There were no example and practice items for the 3-feature Combined test items.

For the 2, 2-feature Separated condition example items, participants were told they would see two patterns (the experimenter pointed to the two matrices), and that each pattern would show one part of the answer that should be drawn in the box below. They were then guided through each feature of the 2, 2-feature example items in the same way as for the Combined condition and were given feedback on their performance. They then completed 5, 2-feature practice items by themselves without feedback. There were no practice and example items for the 3-feature Separated test items.

Participants were made aware of the connection between the two conditions. If they completed the Combined condition first, they were told the Separated condition would be similar, but with one change—multiple patterns would be presented instead of one, with each showing part of the answer. Participants who completed the Separated condition first were told the Combined condition would be similar but that a single matrix would contain shapes with two or three parts that needed to be drawn.

Each task item was presented on an A4 page in landscape orientation. Participants were given unlimited time to solve each item. Time to first stroke and overall drawing time were recorded using a stopwatch. For both the Separated and Combined tasks, the proportion of correct features was scored. This was calculated by scoring the number of features correct per item (out of 3), and then summing the features correct across items to provide a total number of features correct (out of a total of 30; 10 items with a possible 3 features per item). This was converted to a proportion correct (total correct/30). We chose to use partial credit scoring, giving credit for partially correct answers, because it makes more sense from a test-theory perspective. Using an absolute scoring method (e.g., only giving credit for whole items that were correct) would have meant discarding information that could be used to distinguish among individuals’ performance.

### Analysis plan

An analysis plan for this and related studies was pre-registered on AsPredicted (#13338) prior to data collection. This states that the analyses relevant to the central research question posed in this study (does segmentation facilitate children’s performance on a modified matrix reasoning task and is there a relationship with age and fluid reasoning ability) would include: (a) general linear models (GLM) to predict proportion of correct responses by condition (Combined and Separated) and fluid reasoning ability, and (b) correlation and regression analyses to test associations between cognitive segmentation and age. The primary analyses followed these steps.

Additional exploratory analyses not stated in the pre-registration were conducted to replicate the analyses of Duncan et al. (2017) in their study with adults. These were run to test whether the segmentation benefit depended on fluid reasoning (e.g., do children with lower fluid reasoning abilities benefit more from segmentation than those with higher fluid reasoning skills). To this end, a GLM was run with Condition as a within-subjects factor and mean-centred fluid reasoning as a between-subjects variable. The Condition × fluid reasoning interaction term was also included in the model. Further analyses were conducted to test whether there were any differential effects of age across the different conditions of the modified matrix reasoning task: a GLM was run with Condition as the within-subjects variable, mean-centred age as the between-subjects variable, and the Age × Condition interaction term. Order effects were also tested to explore whether completing the Separated condition first enhanced performance in the Combined condition. Simple order effects were tested using a GLM with Condition as within-subjects variable and task order as the between-subjects variable. To test whether any beneficial effect of receiving the Separated condition first was greater for younger children or those with low fluid reasoning, a GLM was run with Condition as a within-subjects factor, task order as a between-subjects factor, and mean-centred fluid reasoning and mean-centred age as between-subjects covariates. The proportion of correct score from the Cattell-based Fluid Reasoning task was entered as the primary measure of fluid reasoning in these analyses, because Cattell’s Culture Fair Test had been used in Duncan et al. (2017). As this measure was not age-standardised, the analyses were rerun using an age-standardised child measure of IQ, the Leiter-3 (Roid et al., 2013).

A final set of exploratory analyses investigated drawing errors and drawing times. This provided additional insight into the problem-solving performance. For each feature, errors were classified into one of five categories: wrong alternative; omission of a feature; other drawing error, item not attempted, or copying the C-term. Researcher (SO) coded the types of errors made on each feature for all participants. Based on the children’s drawing, S.O. was unsure of error type assignments for 26 participants. D.J.M. independently scored the data for those 26 participants. S.O. and D.J.M. discussed their error code assignments and where there were disagreements, J.H. decided on the final error assignment. “Wrong alternative” errors occurred when the participant drew the alternative feature that was presented in the matrix but was not the correct response feature. Participants who either forgot, or failed to draw, all three features committed an “omission of a feature” error. A “drawing error” referred to instances when a participant drew a feature that did not appear in the matrix, or applied an extra transformation such as reflection. “Item not attempted” errors occurred when participants refused to attempt any part of the item. “Copying the C-term” errors occurred when participants copied all features of the item in the C cell of the matrix. The number of each type of error was calculated as follows: each incorrect answer was categorised as one of the five types of errors. The number of each type of error made was calculated for both the Combined and Separated conditions. Error data were missing for four participants. Two repeated measures ANOVA examined how the number of errors varied, according to error type, within each condition (Combined and Separated). A repeated measures ANCOVA was run to examine whether the proportion of each error type varies with condition, fluid reasoning, and age. Additional measures explored were the time to the first drawing stroke and overall drawing time, for each condition.

## Results

### Cognitive segmentation and fluid intelligence

Performance was higher in the Separated (*M* = 0.89; *SD* = 0.16) than the Combined condition (*M* = 0.56; *SD* = 0.19). Mean performance was close to ceiling in the Separated condition. Average proportion correct on the Cattell-based fluid reasoning task was *M* = 0.52; *SD* = 0.14.

**Figure 2.**
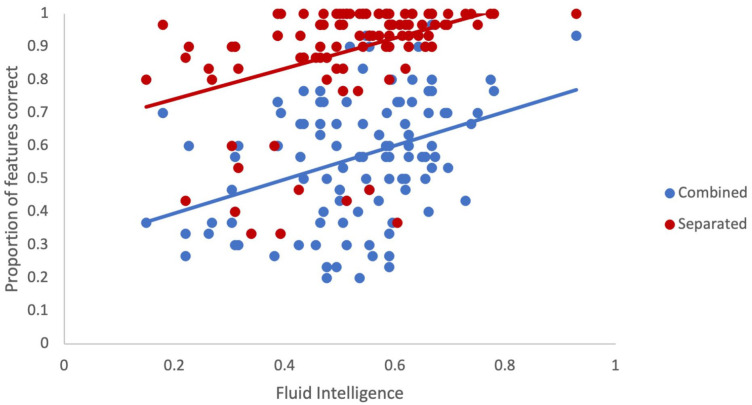
Association between performance in the combined and separated conditions and fluid reasoning (Cattell). *R*^2^ values come from simple correlations per condition (combined: *R*^2^ = .16; separated: *R*^2^ = .14).

A GLM predicting proportion of correct features from Condition (Combined and Separated) and mean-centred fluid reasoning revealed a significant main effect of Condition, *F* (1, 113) = 379.02, *p* < .001, η_*p*_^2^ = .77 (see Figure 2), and fluid reasoning, *F* (1, 113) = 29.18, *p* < .001, η_*p*_^2^ = .205. Children performed better in the Separated condition, and those with higher fluid reasoning scores achieved better scores in both the Separated and Combined conditions relative to those with lower fluid reasoning scores. There was no interaction between Condition and fluid reasoning, *F* (1, 113) = 0.14, *p* = .71, η_*p*_^2^ = .001, hence there was no evidence that the Separated condition was more helpful for children with lower fluid reasoning scores. As our Cattell-based fluid reasoning task was not age-standardised, an additional GLM was run to test the age × IQ × condition interaction. The main effects of Condition, *F* (1, 111) = 349.33, *p* < .001, η_*p*_^2^ = .76, and fluid reasoning, *F* (1, 111) = 17.92, *p* < .001, η_*p*_^2^ = .12, remained, but there was no main effect of age, *F* (1, 111) = 3.83, *p* = .053, η_*p*_^2^ = .033, and crucially the Condition × fluid reasoning × age interaction was not significant, *F* (1, 111) = 0.60, *p* = .44, η_*p*_^2^ = .005.

The Cattell-based fluid reasoning task included in these analyses was similar to the one employed by Duncan et al. (2017) in their study with adults. To further control for the fact that this task was not standardised for age, the analyses were rerun using the age-standardised child measure of IQ, the Leiter International Performance Scale-Revised (Leiter-3: Roid et al., 2013). The same pattern of results emerged using this scale. There was a significant main effect of Condition, *F* (1, 113) = 379.15, *p* < .001, η_*p*_^2^ = .77, and Leiter, *F* (1, 113) = 44.65, *p* < .001, η_*p*_^2^ = .283, but there was no interaction between Condition and Leiter, *F* (1, 113) = 0.20, *p* = .66, η_*p*_^2^ = .002.

### Cognitive segmentation and age

**Figure 3.**
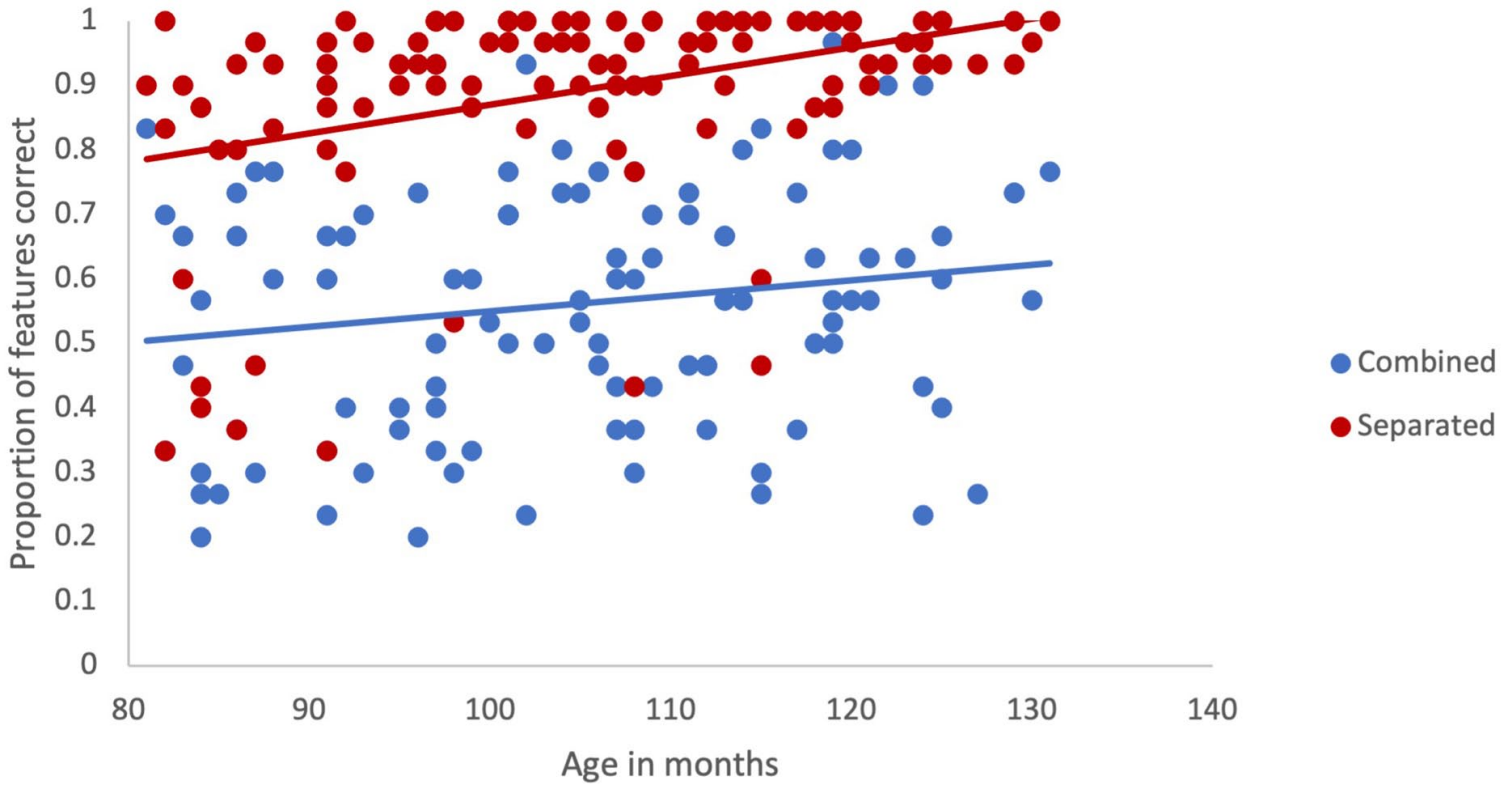
Association between performance in the combined and separated conditions and age in months. *R*^2^ values come from simple correlations per condition (combined: *R*^2^ = .14; separated: *R*^2^ = .03).

A GLM predicting proportion of correct features from Condition (Combined and Separated) and mean-centred age revealed a significant main effect of age, *F* (1, 113) = 11.73, *p* < .001, η_*p*_^2^ = .094. Children performed better in the Separated condition, but there was no interaction between Condition and Age, *F* (1, 113) = 2.63, *p* = .11, η_*p*_^2^ = .023, hence no evidence that the Separated condition was more helpful for younger or older children (Figure 3).

### Order effects

To test whether there were practice effects associated with completing the Separated condition first, order effects were investigated. Performance was independent of condition order, *F* (1, 113) = 0.01, *p* = .94, η_*p*_^2^ = .000, and there was no condition × order interaction, *F* (1, 113) = 0.55, *p* = .46, η_*p*_^2^ = .005. These conclusions held when fluid reasoning (indexed by either the Cattell-based or Leiter measures) and age were added as covariates. For all higher-order interactions of condition × order with these covariates, *F* < 0.97, *p* > .76, η_*p*_^2^ < .009. Therefore, there was no evidence that task order effects might depend on fluid reasoning ability or age.

### Exploratory error analysis

#### Error types

Our free-response format allowed us to analyse variability in the types of errors that children produced. Two GLMs were first run to examine how the number of errors varied according to error type within each condition (Combined and Separated). Then, to understand whether the types of errors participants made varied with condition, age, and fluid reasoning, a third model was run with condition added as a within-subject factor and age and fluid reasoning as between-subject covariates.

**Figure 4.**
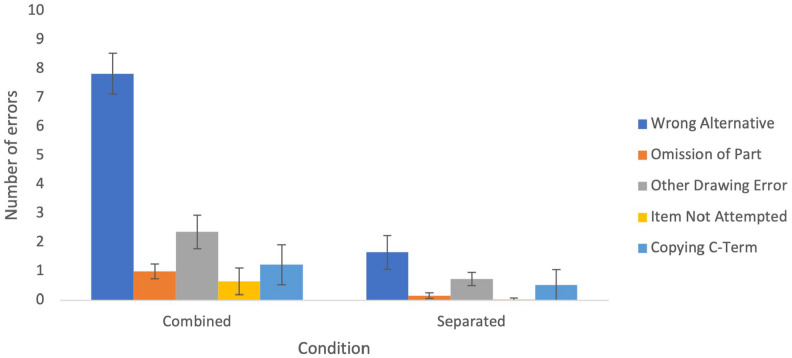
Mean number of errors of each type, within the Combined and Separated conditions. Error bars are 95% between-subject confidence intervals.

Within the Combined condition, Mauchly’s test indicated that the assumption of sphericity had been violated, χ^2^(9) = 98.76, *p* < .001, therefore the degrees of freedom were corrected using Greenhouse–Geisser estimates of sphericity (ε = .77). The results revealed a significant main effect of Error Type, *F*(3.07, 337.33) = 101.26, *p* < .001, η_*p*_^2^ = .479. Post hoc analysis with Bonferroni adjustment revealed that the frequency of “wrong alternative” errors (*M* = 7.83, *SD* = 3.78) was significantly higher than all other error types: “omission of part” (*M* = 0.99, *SD* = 1.35); “other drawing error” (*M* = 2.36, *SD* = 3.14); “item not attempted” (*M* = 0.65, *SD* = 2.44); and “copying the C-term” (*M* = 1.22, *SD* = 3.71). The number of “other drawing errors” was significantly higher than “omission of part” and “item not attempted” errors. No other pairwise differences were significant (see Figure 4).

Within the Separated condition, Mauchly’s test again indicated nonsphericity, χ^2^(9) = 223.57, *p* < .001, therefore the degrees of freedom were corrected using Greenhouse–Geisser estimates of sphericity (ε = .55). As with Combined features, there was a significant main effect of Drawing Error, *F*(2.21, 243.06) = 11.98, *p* < .001, η_*p*_^2^ = .098. The same pattern of results also emerged for post hoc analysis (with Bonferroni adjustment). “Wrong alternative” errors (*M* = 1.66, *SD* = 3.13) were significantly higher than all other error types: “omission of part” (*M* = 0.16, *SD* = 0.48); “other drawing error” (*M* = 0.73, *SD* = 1.24); “item not attempted” (*M* = 0.03, *SD* = 0.28); and “copying the C-term” (0.52, *SD* = 2.87). Again, “other drawing errors” were higher than “omission of part,” ‘item not attempted,’ and “copying the C-term.” The number of “other drawing errors” was significantly higher than “omission of part” and “item not attempted.” No other pairwise differences were significant (see Figure 4).

A repeated-measures ANCOVA tested whether the distribution of error types depended on Condition, Cattell-based Fluid Reasoning, or Age. For this analysis we were again interested in the main effect of Error Type, as well as its interaction with the other factors. There was a significant main effect of Error Type, *F*(2.89, 309.52) = 100.35, *p* < .001, η_*p*_^2^ = .484, and a significant Error Type × Condition interaction, *F*(2.77, 296.08) = 42.95, *p* < .001, η_*p*_^2^ = .286. The interaction indicates that although the Separated condition reduced errors of every type, the most common “wrong alternative” errors were especially reduced. There was no significant Error Type × Fluid Reasoning interaction, *F*(2.89, 309.52) = 1.96, *p* = .12, η_*p*_^2^ = .018, and no Error Type × Age interaction, *F*(2.89, 309.52) = 2.31, *p* = .08 η_*p*_^2^ = .021. No higher-order interactions were significant, *F* < 2.22, *p* > .09, η_*p*_^2^ < .020.

This analysis was repeated substituting the Cattell-based fluid reasoning measure with the Leiter. The pattern of results was unchanged. There was a significant main effect of Error Type, *F*(2.91, 311.51) = 110.45, *p* < .001, η_*p*_^2^ = .508, and a significant Error Type × Condition interaction, *F*(2.78, 297.73) = 44.94, *p* < .001, η_*p*_^2^ = .296. There was no significant Error Type × Fluid Reasoning interaction, *F*(2.91, 311.51) = 1.99, *p* = .11 η_*p*_^2^ = .018, nor an Error Type × Age interaction, *F*(2.91, 311.51) = 1.90, *p* = .13 η_*p*_^2^ = .02, nor any higher-order interactions (*F* < 1.98, *p* > .12, η_*p*_^2^ < .018).

#### Drawing time

Drawing times revealed that the time from problem presentation to first stroke was significantly longer in the Combined (*M* = 11.47 s, *SD* = 5.71) than the Separated condition (*M* = 6.79 s, *SD* = 2.79), *Z* = –7.96, *p* < .001, as for adults (Duncan et al., 2017). The total time spent drawing (from first to last stroke) was significantly longer in the Separated condition (*M* = 23.68 s, *SD* = 7.39) compared with the Combined condition (*M* = 17.71 s, *SD* = 6.76), *Z* = –7.29, *p* < .001), as expected, because children were more likely to omit a part in the Combined condition.

## Discussion

Fluid reasoning skills are highly predictive of success in a wide range of areas (e.g. Deary, 2012; Duncan, 2013; Postlethwaite, 2011). In adults, performance on matrix reasoning tasks relies on the ability to segment complex problems into smaller separately attended parts, a skill referred to as cognitive segmentation (Duncan et al., 2017). Eye-tracking studies indicate that children may fail to spontaneously segment in that they fail to focus on the A:B relation before moving to C (Thibaut & French, 2016). The current study is the first replication of Duncan et al.’s (2017) study, which involved the development of a child-friendly version of their segmentation task. Like adults in Duncan et al.’s (2017) study, children were given two versions of a modified matrix reasoning task. Both versions reduced demands on other cognitive skills such as working memory and processing speed, but one version additionally made it trivial to separate the problems into smaller parts. As with adults, performance on both tasks was related to fluid intelligence, and better in the condition in which the problems were separated into component parts. Unlike adults, children with lower fluid intelligence did not benefit more in the separated condition than children with higher fluid intelligence. Older children performed better than younger children in both conditions, but there was no evidence that breaking problems down was more beneficial for younger than older children. These results are discussed in turn below.

Duncan et al. (2017) linked adult problem-solving skills to the principle of compositionality and the attentional control functions of the frontal and parietal cortex (Duncan, 2013; Duncan et al., 2020; Tschentscher et al., 2017) when they found that performance on a modified matrix reasoning task could be improved by breaking problems down into smaller parts. Our data reveal that the same is true for children. When the working memory and processing speed demands of traditional matrix reasoning problems were minimised by removing time constraints and allowing children to draw the answers, children’s performance in the Combined condition was worse than in the Separated condition in which the problem was broken down into its component features across three separate cells. This suggests that cognitive segmentation is a critical component of solving matrix reasoning tasks in children, as it is for adults. It is important to note that while we aimed to minimise demands on working memory and processing speed, we cannot rule out the possibility that these demands may differ across the two task conditions. Future work is needed to quantify the extent to which working memory, processing speed, and cognitive segmentation make independent contributions to matrix reasoning task performance.

Performance in both conditions was linked to fluid intelligence, as expected, both because nonverbal reasoning is associated with performance on most cognitive tasks (Deary, 2012; Jensen, 1998; Kovacs & Conway, 2016; Spearman, 1927), and because performance on the fluid intelligence tasks requires focussed attention on the right things at the right time (i.e., segmentation, Duncan et al., 2017). Duncan et al. (2017) found that adults with a low IQ benefitted more from the Separated condition than adults with a high IQ. Unlike adults, there was no interaction between performance in the two conditions and children’s fluid intelligence, suggesting that children with lower fluid intelligence did not benefit more from the problems being broken down for them. One explanation is that all 6 to 10-year-old children perform like adults with lower fluid intelligence and struggle to spontaneously segment problems in the Combined condition. In contrast to children and adults with lower fluid intelligence, adults with higher IQ might spontaneously segment, leading to much improved performance in the Combined condition, and accordingly less benefit in the Separated condition.

There was a main effect of age: older children performed better than younger children in both the Separated and Combined conditions. Age-related improvements in the Combined condition in our sample of children aged 6+ are consistent with widely reported developmental improvements in reasoning (Christie & Gentner, 2014; Ferrer et al., 2009; McArdle et al., 2002; Richland et al., 2006; Starr et al., 2018; Sternberg & Rifkin, 1979) that are more pronounced after the relational shift (Gentner, 1988; Rattermann & Gentner, 1998). These developments are likely related to both increases in children’s knowledge about conceptual similarities (e.g., Gentner, 1988; Goswami & Brown, 1990), and developmental increases in other cognitive abilities vital for performance on analogical reasoning tasks (e.g., Morrison et al., 2011; Richland et al., 2006; Thibaut et al., 2010). There was no interaction between age and condition, suggesting that age-related improvements in the Separated condition are unlikely to reflect an increase in the ability to break problems down. They may instead be driven by other factors such as an increased ability to understand the task instructions, relational reasoning ability, or age-related changes in attentional focus or motivation.

As children get older they adopt a more adult-like approach to solving matrix problems focussing on the A:B relation prior to moving to C (Starr et al., 2018). It is therefore perhaps surprising that guiding children’s search strategies by separating the problems was not more useful for younger children. There are several reasons why this might be the case. First, the data presented are cross-sectional. Longitudinal data might be more sensitive to subtle age effects. Second, the age range included (6–10 years) might be too narrow to detect cross-sectional differences. Relatively few children towards the far ends of the age distribution may have also contributed to low sensitivity to age-related differences. And finally, within the age range studied, the ability to spontaneously segment may not have started to develop or may be masked by concurrent development of analogical reasoning ability. A shift to basing responses on relational similarities rather than perceptual matches occurs around age 5 years (Gentner & Ratterman, 1991; Goswami, 1992). If the younger children are yet to fully understand matrix relationships, they would fail whether the features were segmented or not, and so the potential for a segmentation benefit would increase with age as analogical reasoning develops. This could then counteract a decreasing segmentation benefit with age due to overlapping development of spontaneous segmentation ability.

We speculated that completing the Separated condition first might benefit performance in the Combined condition by providing a useful strategy to decompose the problems, and practice in applying it. However, just as with adults, children’s performance in the Combined condition did not benefit more from completing the Separated condition first. As suggested by Duncan et al. (2017), the instructions and practice trials for both conditions may have minimised order effects because they emphasised applying the same procedure to both conditions—focussing on one part after another—and because participants were aware of the connection between the conditions. In other words, although instructed and encouraged to break a combined problem into parts, both children and adults with low IQ fail to do so effectively and are not helped by having seen and briefly practised such decomposition.

As in Duncan et al.’s (2017) study, the majority of errors in both Combined and Separated conditions were “wrong alternatives,” that is, drawing the alternative feature that was presented in the matrix, but was not the correct response feature. This might suggest that the children were easily confused when selecting which features to attend and select from cells A, B, or C, and is consistent with the broader literature on children’s problem-solving skills, suggesting that children do not systematically orient and organise their searches across the cells in matrix reasoning problems and analogical reasoning more generally (Chen et al., 2016; Glady et al., 2012, 2017; Starr et al., 2018; Thibaut & French, 2011; Vendetti et al., 2017). While the Separated format reduced errors of all types, and did not induce a qualitatively different pattern of errors, it appeared particularly beneficial in reducing the most common “wrong alternative” errors.

Finally, we note that while the free-drawing response mode allowed us to examine specific error types, and aimed to minimise demands on working memory, and processing speed that might be associated with more typical multiple choice paradigms, we did not attempt to compare different response modes in this study. Previous research has shown both similarities and differences in the impact of constructive versus multiple-choice response modes on children’s matrix reasoning performance (Stevenson et al., 2016). It remains to be seen whether the benefit of the separated format in aiding cognitive segmentation generalises across response modes.

## Conclusion

This study demonstrates that cognitive segmentation is a critical component of complex problem-solving in children, as it is for adults. By forcing children to focus their attention on separate parts of a complex visual problem, their performance can be dramatically improved. This is akin to scaffolding children’s behaviour by dividing complex tasks into simpler steps, and guiding attention to each in term, which has long been regarded as an effective way to aid children’s learning and development (Duncan et al., 2021; Neitzel & Stright, 2003; Vygotsky, 1978; Wood et al., 1976). Interestingly, cognitive segmentation appears to be equally beneficial to all children aged 6 to 10 years, with no greater or lesser effects for children with lower or higher IQs or of different ages within this range. These data underscore the importance of breaking complex problems down in the classroom, for children of all abilities, and restructuring multi-step tasks into separate independent steps to support children’s learning and classroom success.

## Supplemental Material

sj-pdf-1-qjp-10.1177_17470218221116054 – Supplemental material for Cognitive segmentation and fluid reasoning in childhood Click here for additional data file. Supplemental material, sj-pdf-1-qjp-10.1177_17470218221116054 for Cognitive segmentation and fluid reasoning in childhood by Sinéad O’Brien, Daniel J Mitchell, John Duncan and Joni Holmes in Quarterly Journal of Experimental Psychology
