# Supplementary material for: Cognitive segmentation and fluid reasoning in childhood
Source: Q J Exp Psychol (Hove). 2022 Aug 24;76(6):1431–44. doi: 10.1177/17470218221116054 (PMC7614553; doi:10.1177/17470218221116054)
Supplement: sj-pdf-1-qjp-10.1177_17470218221116054 – Supplemental material for Cognitive segmentation and fluid reasoning in childhood [file sj-pdf-1-qjp-10.1177_17470218221116054.pdf]

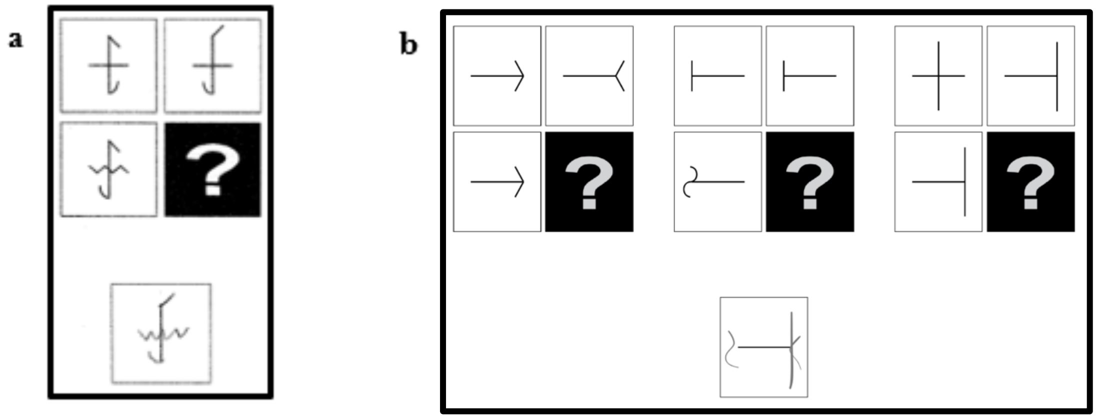

Figure 1: Examples of wrong alternative errors (a) 3-feature Combined (b) 3-feature Separated.

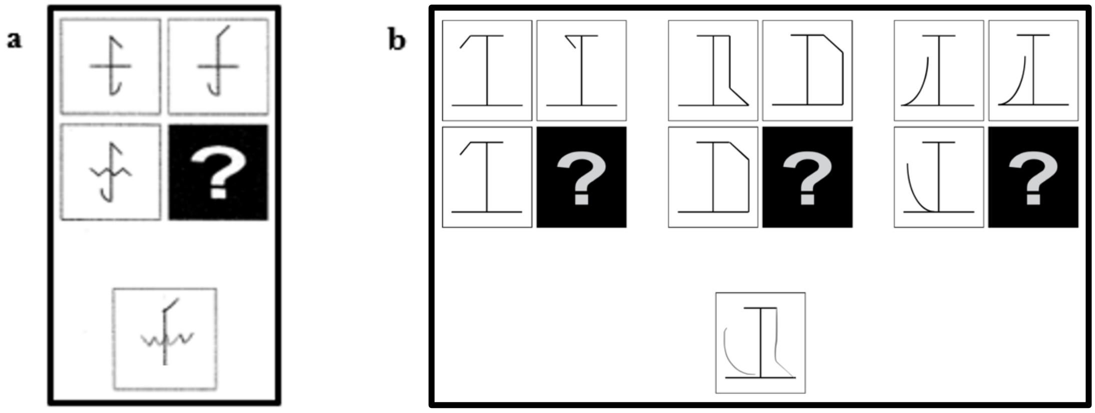

Figure 2: Examples of omission of part errors (a) 3-feature Combined (b) 3-feature Separated.

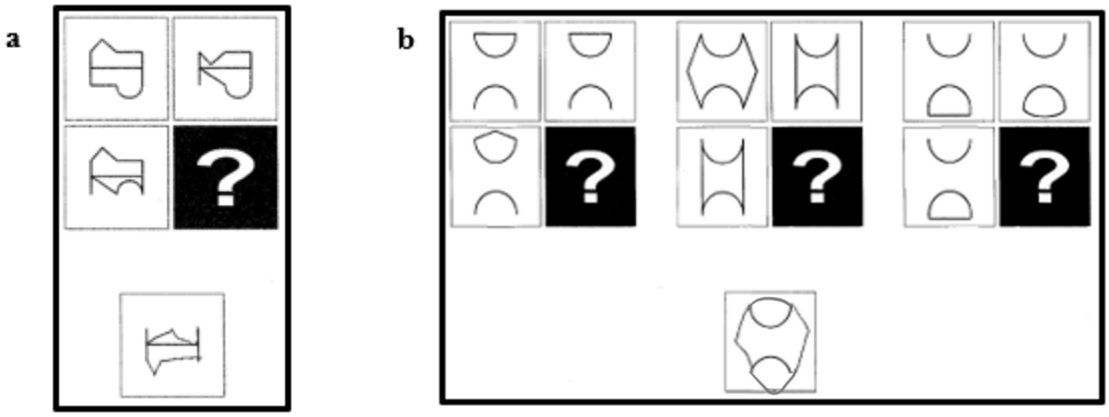

Figure 3: Examples of other drawing errors (a) 3-feature Combined (b) 3-feature Separated

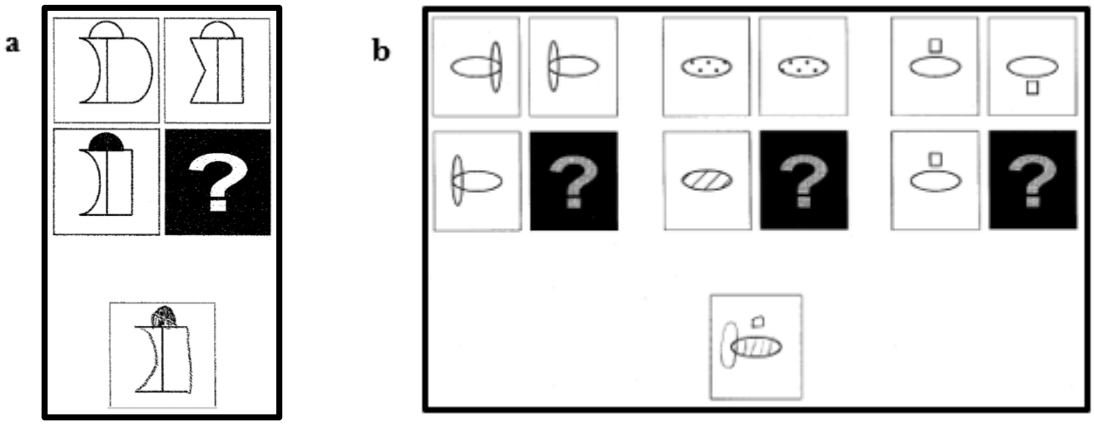

Figure 4: Example of copying the c-term errors (a) 3-feature Combined (b) 3-feature Separated.
